# Clinical suitability of intranasal delivery of M2 macrophage soluble factors in patients with post-COVID olfactory disorders

**DOI:** 10.1192/j.eurpsy.2024.255

**Published:** 2024-08-27

**Authors:** E. Markova, E. Shevela, M. Davydova, I. Meledina, A. Ostanin, V. Kozlov, E. Chernykh

**Affiliations:** ^1^State Research Institute of Fundamental and Clinical Immunology, Novosibirsk, Russian Federation

## Abstract

**Introduction:**

SARS-CoV virus showed transneuronal penetration through the olfactory bulb resulting in the rapid intracranial spread. So, olfactory dysfunction is an early marker of COVID-19 infection. However, individuals may develop chronic olfactory impairment for more than six months in 1–10% of cases.

**Objectives:**

The study’s objective was to evaluate the efficacy and safety of intranasal immunotherapy using bioactive substances produced by M2 macrophages for the treatment of people with long-term post-COVID-19 hyposmia.

**Methods:**

Seven individuals with long-term persistent hyposmia (7 to 24 months), associated with PCR-confirmed coronavirus infection were evaluated for olfactory function at baseline, one, and six to twelve months after therapy.

**Results:**

The intranasal inhalation of M2 macrophage conditioned medium (one time per day for 28-30 days) was well tolerated. Furthermore, olfactometry demonstrated that the patients restored their capacity to perceive (Kruskal-Wallis H test 14.123, p = 0.0009) and recognize odors (H = 11.674, p = 0.0029). In addition, the subjective evaluation of smell significantly improved (H = 11.935, p = 0.0026). At the 6- to 12-month follow-up, the majority of patients (5/7) reported extremely high levels of satisfaction with the outcomes, and the remaining two patients also felt generally positive about the therapy’s success.

**Conclusions:**

Overall, our study showed that the use of intranasal inhalations as a method of delivering bioactive factors and the conditioned medium of M2 macrophages as a therapeutic agent are both safe, well tolerated and, according to preliminary data, clinically effective in the treatment of patients with long-term post-COVID-19 hyposmia.

**Disclosure of Interest:**

None Declared

